# Thromboelastography‐Guided Laparoscopic Splenectomy in a Patient With Immune Thrombocytopenia and Recent Drug‐Eluting Stent Placement: A Case Report

**DOI:** 10.1155/cria/9911293

**Published:** 2026-07-31

**Authors:** Josue Gaona, Janki Patel

**Affiliations:** ^1^ School of Medicine, University of Texas at Tyler, Tyler, Texas, USA, uttyler.edu; ^2^ Department of Anesthesiology and Perioperative Medicine, University of Texas at Tyler Health Science Center, Tyler, Texas, USA

**Keywords:** drug-eluting stent, dual antiplatelet therapy, immune thrombocytopenia, perioperative management, splenectomy, thromboelastography

## Abstract

**Background:**

Perioperative management of patients with immune thrombocytopenia (ITP) is particularly challenging when complicated by recent coronary stenting requiring dual antiplatelet therapy (DAPT), due to competing risks of bleeding and thrombosis.

**Case Presentation:**

We report the case of an 80‐year‐old male with refractory ITP and recent drug‐eluting stent placement who underwent elective laparoscopic splenectomy. Although preoperative optimization with corticosteroids and intravenous immunoglobulin increased platelet count from < 20 × 10^9^/L to 177 × 10^9^/L, functional hemostatic adequacy remained uncertain given concurrent ITP and ongoing antiplatelet therapy. Thromboelastography (TEG) was performed using the TEG 6s system with a Global Hemostasis Cartridge. Standard TEG parameters demonstrated preserved clot initiation, formation, and strength across multiple channels, supporting progression to surgery. Laparoscopic splenectomy was completed under general anesthesia without complications or need for transfusion. The patient recovered on the general surgical ward and was discharged on Postoperative Day 3 with a platelet count of 139 × 10^9^/L. Platelet count 2 weeks postoperatively was 343 × 10^9^/L.

**Conclusion:**

This case highlights the utility of TEG as a tool to guide perioperative decision‐making in patients with ITP receiving antiplatelet therapy. By providing a functional assessment of coagulation, TEG can help individualize perioperative management in patients with complex hematologic conditions, facilitating safe surgical intervention.

## 1. Introduction

Immune thrombocytopenia (ITP) is an acquired autoimmune disorder characterized by isolated thrombocytopenia resulting from antibody‐mediated destruction of platelets. The clinical presentation of ITP varies widely, ranging from asymptomatic thrombocytopenia to mucocutaneous bleeding and, rarely, life‐threatening hemorrhage [1]. In adults, ITP tends to be chronic and relapsing, and a significant number of patients do not achieve sustained remission with first‐line therapy alone [2]. Splenectomy remains one of the most effective second‐line treatments, achieving long‐term remission in 60%–70% of cases. However, this treatment option is generally deferred for at least 12 months after diagnosis due to the potential for spontaneous remission and the risks associated with surgery [3].

When splenectomy is pursued, careful perioperative planning is essential. Patients with ITP present unique perioperative challenges, particularly in the presence of comorbidities that further compromise hemostasis. In patients with recent drug‐eluting stent (DES) placement, the need for dual antiplatelet therapy (DAPT) to prevent stent thrombosis introduces an additional layer of complexity. These patients face increased perioperative bleeding risk from both thrombocytopenia and antiplatelet‐induced platelet dysfunction, while simultaneously facing the risk of stent thrombosis from interruption of DAPT for surgery [4]. Successful surgical outcomes depend on careful navigation of these competing risks.

Standard evaluation of bleeding risk often centers on platelet count. However, platelet count alone may be an insufficient predictor of bleeding tendency, especially in ITP, where platelet function is often impaired [5]. Furthermore, pharmacologic inhibition of platelet aggregation by antiplatelet agents is not reflected by platelet count. As a result, functional assays such as thromboelastography (TEG) have been proposed as a means of more comprehensively evaluating hemostatic competence in this setting.

TEG is a point‐of‐care, whole‐blood viscoelastic assay that provides a global, real‐time assessment of hemostasis, including clot initiation, formation, strength, and lysis. In the TEG 6s system, multiple channels are run simultaneously from a single blood sample, each reflecting distinct aspects of coagulation. While standard TEG parameters have been shown to correlate with bleeding risk in thrombocytopenic patients better than platelet count alone [6, 7], they do not directly measure pharmacodynamic platelet inhibition from aspirin or P2Y12 antagonists. PlateletMapping, a TEG‐based assay, is designed specifically to quantify the degree of antiplatelet drug effect. This distinction is clinically important, as the utility of standard TEG parameters in patients on DAPT is limited without a concomitant platelet function assessment.

We present a case in which preoperative TEG 6s testing was incorporated into perioperative decision‐making for an elderly patient with refractory ITP and recent DES placement undergoing laparoscopic splenectomy. This report highlights both the value and the practical limitations of this approach in a clinically complex, high‐risk patient.

Written informed consent for publication of this case report was obtained from the patient.

## 2. Case Presentation

An 80‐year‐old Caucasian male with a complex medical history including coronary artery disease, paroxysmal atrial fibrillation, cerebrovascular accident, deep vein thrombosis, recent DES placement following a STEMI, and chronic refractory ITP was referred for elective laparoscopic splenectomy. Over the prior year, the patient had experienced multiple relapses despite various first‐ and second‐line therapies, including high‐dose dexamethasone, weekly romiplostim injections, daily avatrombopag tablets, and multiple intravenous immunoglobulin (IVIG) infusions. Ultimately, splenectomy was recommended.

Approximately 4 months prior to the scheduled procedure, the patient underwent DES placement and was initiated on DAPT with aspirin 81 mg and clopidogrel 75 mg daily. In the weeks leading up to surgery, the case was further complicated by an NSTEMI 5 days prior to the original operative date, necessitating a 1‐month postponement. As the new operative date approached, cardiology advised discontinuing clopidogrel 5–7 days preoperatively while continuing aspirin to mitigate the risk of stent thrombosis.

One week before surgery, the patient’s platelet count remained persistently below 20 × 10^9^/L. Due to this critical thrombocytopenia, oncology initiated prednisone 80 mg daily. To further optimize platelet count, three infusions of IVIG (60 g, 80 g, and 30 g) were administered beginning five days preoperatively. On the day of surgery, his platelet count had increased to 177 × 10^9^/L.

Despite this improvement, the care team recognized that platelet count alone did not fully characterize hemostatic risk in the context of ITP and ongoing aspirin therapy. Accordingly, preoperative TEG was performed using the TEG 6s system (Haemonetics, Boston, MA) with a Citrated Kaolin (CK), Citrated Kaolin with Heparinase (CKH), Citrated RapidTEG (CRT), and Citrated Functional Fibrinogen (CFF) Global Hemostasis Cartridge. Parameters from each channel are summarized in Table 1. Briefly: the CK channel assesses overall clot formation and strength reflecting combined platelet and fibrinogen contributions (R: clot initiation time; K: clot formation rate; angle: rate of fibrin cross‐linking; MA: maximum clot strength); the CRT channel provides MA through accelerated coagulation using kaolin and tissue factor; the CKH channel uses heparinase to rule out a heparin contribution to coagulopathy; and the CFF channel isolates fibrin‐specific clot strength and provides a functional fibrinogen level (FLEV).

**TABLE 1:** Preoperative TEG 6s results (Citrated K, KH, RT, and FF Global Hemostasis Cartridge).

Channel	Parameter	Value	Reference range	Clinical meaning
CK	R (min)	5.7	4.6–9.1	Time to initial clot formation
CK	K (min)	0.9	0.8–2.1	Rate of clot development
CK	Angle (°)	77.6	63–78	Speed of fibrin/platelet cross‐linking
CK	MA (mm)	66.5	52–69	Overall clot strength (platelet + fibrinogen)
CRT	MA (mm)	68.3	52–70	Clot strength with accelerated coagulation
CKH	R (min)	6.0	4.3–8.3	Heparinase channel; rules out heparin effect
CFF	MA (mm)	34.6 ↑	15–32	Fibrin‐specific clot strength (elevated)
CFF	FLEV (mg/dL)	631.4 ↑	278–581	Functional fibrinogen level (elevated)

*Note:* CKH: Citrated Kaolin with Heparinase; R: reaction time; K: clot kinetics; FLEV: functional fibrinogen level. ↑ above reference range.

Abbreviations: CFF: Citrated Functional Fibrinogen; CK: Citrated Kaolin; CRT: Citrated RapidTEG; MA: maximum amplitude.

CK channel values were within normal limits (R: 5.7 min, K: 0.9 min, angle: 77.6°, MA: 66.5 mm), as were CRT‐MA (68.3 mm) and CKH‐R (6.0 min). The CFF channel revealed mildly elevated CFF‐MA (34.6 mm; reference: 15–32 mm) and CFF‐FLEV (631.4 mg/dL; reference: 278–581 mg/dL), consistent with an elevated FLEV most likely reflecting an acute‐phase response following recent IVIG administration and corticosteroid therapy. These findings indicated preserved and, in some respects, augmented hemostatic function, providing the care team with confidence in proceeding with surgery.

An attempt was made to obtain a more specific assessment of antiplatelet drug effect. PlateletMapping was ordered but could not be performed, as the required cartridge was unavailable. A platelet function analyzer (PFA) was ordered as an alternative but was ultimately contraindicated due to the patient’s hematocrit of 32.5%, which was below the minimum threshold of 35% required for valid PFA results. As a result, the degree of residual platelet inhibition from aspirin could not be directly quantified, and perioperative management proceeded based on clinical judgment informed by the standard TEG 6s parameters, the platelet count of 177 × 10^9^/L, the 7‐day clopidogrel hold, and multidisciplinary input from cardiology, hematology, and surgery.

The patient underwent general anesthesia with stress‐dose corticosteroids. Given his significant cardiovascular comorbidities, invasive hemodynamic monitoring was employed. Laparoscopic splenectomy was completed uneventfully, with an estimated blood loss of 75 mL and no need for intraoperative or postoperative transfusion. No intraoperative TEG was performed, as the clinical course did not prompt additional hemostatic assessment. The patient was transferred to the general surgical ward postoperatively.

Postoperative CBC values are summarized in Table 2. The patient maintained stable hemoglobin and hematocrit throughout the postoperative period. Platelet count declined transiently to 118 × 10^9^/L on Postoperative Day 2, consistent with expected post‐splenectomy hemodynamic redistribution, before recovering to 139 × 10^9^/L at discharge on Postoperative Day 3. He was discharged in stable condition with instructions to resume clopidogrel. Subsequent outpatient follow‐up demonstrated continued hematologic and cardiovascular stability, with a platelet count of 343 × 10^9^/L 2 weeks postoperatively. Hematology noted a favorable ITP response and began tapering corticosteroids. Follow‐up with general surgery and cardiology was unremarkable.

**TABLE 2:** Perioperative laboratory values.

Parameter	Preoperative	POD 1	POD 2	POD 3/discharge
WBC (K/mm^3^)	12.2 ↑	13.6 ↑	14.4 ↑	13.1 ↑
Hemoglobin (g/dL)	10.2 ↓	9.3 ↓	10.2 ↓	10.3 ↓
Hematocrit (%)	32.5 ↓	28.6 ↓	31.9 ↓	32.3 ↓
Platelets (× 10^9^/L)	177	143	118 ↓	139

*Note:* ↑ above reference range; ↓ below reference range. Reference ranges: WBC: 3.8–10.6 K/mm^3^; hemoglobin: 14.0–18.0 g/dL; hematocrit: 38.0%–54.0%; platelets: 130–400 × 10^9^/L.

## 3. Discussion

This case illustrates the perioperative complexity encountered when managing a patient with refractory ITP, recent DES placement, and ongoing aspirin therapy who required elective splenectomy. The coexistence of thrombocytopenia, antiplatelet‐induced platelet dysfunction, and thrombotic risk from recent coronary stenting created a situation in which neither platelet count alone nor standard coagulation testing could adequately characterize hemostatic competence. This case highlights both the potential utility and the important limitations of TEG‐based assessment in this context.

A major challenge in managing ITP lies in its unpredictable bleeding phenotype. Studies have shown that patients with similar degrees of thrombocytopenia can have widely variable bleeding phenotypes, with some experiencing significant bleeding at higher counts and others remaining asymptomatic at lower counts. This variability reflects differences in platelet function and other patient‐specific factors such as age, comorbidities, and prior bleeding history [8]. As a result, platelet count alone provides an incomplete picture of bleeding risk in patients with ITP.

In this case, TEG was used to provide a more functional evaluation of the patient’s coagulation status on the day of surgery. The TEG 6s Global Hemostasis Cartridge generates simultaneous results from four channels, each reflecting distinct aspects of hemostasis. In the CK channel, which assesses overall clot formation and strength through combined platelet and fibrinogen contributions, all parameters were within normal limits. CK‐MA of 66.5 mm, reflecting global clot strength, was within the reference range of 52–69 mm. The CRT‐MA of 68.3 mm, which reflects clot strength under accelerated coagulation, was similarly normal. These values indicated that despite a history of severe thrombocytopenia and recent medical optimization, the patient was capable of generating a clot of normal strength on the day of surgery.

It is important to note that CK‐MA specifically reflects the combined contributions of platelet quantity, platelet function, and fibrinogen to clot strength, while CFF‐MA isolates the fibrin‐specific component. The distinction between these parameters is clinically meaningful: an elevated CFF‐MA, as observed in this patient (34.6 mm; reference: 15–32 mm), does not indicate hypercoagulability per se but rather an increased fibrin contribution to clot strength, likely attributable to elevated fibrinogen in the setting of recent IVIG administration and high‐dose corticosteroid therapy. Similarly, the elevated CFF‐FLEV (631.4 mg/dL) reflected a high FLEV consistent with an acute‐phase response. These findings were interpreted as reassuring rather than alarming, as robust fibrin‐based clot integrity helped offset concerns about platelet function. The *α*‐angle (CK‐angle: 77.6°), reflecting the rate of fibrin cross‐linking and clot propagation, was within normal limits, suggesting unimpaired clot kinetics [9]. CKH‐R (6.0 min) was also normal, confirming that heparin effect did not contribute to any apparent coagulopathy, as no heparin was administered.

Published TEG findings in ITP have generally described reduced MA and altered clot kinetics in the setting of active thrombocytopenia [10, 11]. However, there are limited data specifically describing TEG parameters in ITP patients whose platelet counts have normalized or improved following corticosteroid therapy and/or IVIG. In such patients, it is plausible that the TEG profile normalizes in parallel with platelet count recovery, particularly if platelet function is also restored. In the present case, the normal CK‐MA on the day of surgery, despite the patient’s history of refractory ITP, is consistent with this hypothesis and suggests that the combination of medical optimization and residual platelet function was sufficient to support normal clot strength. Further investigation is needed to characterize TEG parameters specifically in ITP patients who have undergone treatment‐mediated platelet count recovery, as this information would help clinicians better interpret point‐of‐care hemostatic data in this population.

A critical limitation of this case’s hemostatic assessment warrants explicit discussion. Standard TEG parameters, including the CK and CRT channels, do not directly quantify pharmacodynamic platelet inhibition from aspirin or P2Y12 receptor antagonists such as clopidogrel. PlateletMapping, a TEG‐based assay that uses ADP and arachidonic acid activators to quantify residual platelet aggregation in the presence of antiplatelet agents, is the appropriate tool for this purpose. Unfortunately, PlateletMapping could not be performed in this case because the required cartridge was not available. A PFA was subsequently ordered as an alternative platelet function test but could not be performed due to the patient’s hematocrit of 32.5%, below the minimum threshold of 35% required for valid PFA results. As a result, the degree of aspirin‐induced platelet inhibition was never directly quantified, and the clinical team proceeded based on the combination of standard TEG parameters, the 7‐day clopidogrel washout, continuation of aspirin, and multidisciplinary judgment.

This experience underscores an important practical limitation: in institutions where PlateletMapping cartridges may not be routinely stocked, the ability of TEG to inform antiplatelet management is significantly constrained. Anesthesiologists and perioperative physicians should be aware of this gap and plan accordingly, ensuring that appropriate cartridges and alternative assays are available when managing patients on DAPT who require surgery. In situations where direct platelet function assessment is unavailable, perioperative decisions must rely on clinical pharmacology principles, including expected drug washout times and multidisciplinary input.

Preoperative management of DAPT was equally critical. In patients with recent DES placement, interruption of antiplatelet therapy must be carefully weighed against the risk of stent thrombosis. According to the 2023 ACC/AHA guideline for the management of chronic coronary disease, patients undergoing percutaneous coronary intervention with DES placement are generally recommended to receive DAPT for 6 months, followed by single antiplatelet therapy. However, shorter durations of 1–3 months may be considered in patients at high risk of bleeding. In clinical scenarios such as ITP requiring splenectomy, individualized decisions regarding early discontinuation may be warranted [12, 13]. In this case, surgery was performed 4 months after DES placement. Clopidogrel was held for 7 days preoperatively while aspirin was continued, and DAPT was resumed promptly after surgery per cardiology recommendations. This strategy provided adequate protection from thrombosis without compromising surgical safety.

Hematologic and endocrine optimization further supported a successful surgical outcome. IVIG was used to transiently raise the platelet count, an established strategy for refractory ITP when rapid correction is required [14]. In addition, the patient’s chronic corticosteroid use necessitated administration of perioperative stress‐dose steroids to prevent adrenal insufficiency and hemodynamic instability. This measure was particularly important given the patient’s history of cerebrovascular disease within the preceding year, as perioperative hypotension and inadequate steroid replacement have been associated with increased cardiovascular and neurologic complications [15].

Ultimately, this case demonstrates the value of integrating available functional hemostatic testing with clinical pharmacology principles and multidisciplinary coordination when managing surgical patients with complex, overlapping hemostatic challenges. TEG provided objective evidence of adequate clot formation and strength on the day of surgery, which complemented the platelet count and supported a decision to proceed. However, its inability to directly assess antiplatelet drug effect in this case should serve as a reminder that point‐of‐care hemostatic testing must be interpreted within the full clinical context, including the specific assays available, their known limitations, and the clinical pharmacology of the agents in use. Larger studies are needed to define optimal perioperative TEG thresholds and to characterize TEG profiles in ITP patients following medical optimization, but our experience supports a role for TEG as one component of a structured, individualized perioperative hemostatic assessment.

## Author Contributions

Josue Gaona: writing–original draft and investigation.

Janki Patel: supervision; conceptualization; resources; and writing–review and editing.

## Funding

No funding was received for this work.

## Conflicts of Interest

The authors declare no conflicts of interest.

## Data Availability

All data are available within the article. No additional datasets were generated or analyzed for this study.
